# Can Rare Earth Elements Be Considered as Markers of the Varietal and Geographical Origin of Wines?

**DOI:** 10.3390/molecules28114319

**Published:** 2023-05-24

**Authors:** Zaual Temerdashev, Mikhail Bolshov, Aleksey Abakumov, Alexan Khalafyan, Anastasia Kaunova, Alexander Vasilyev, Olga Sheludko, Arsen Ramazanov

**Affiliations:** 1 Analytical Chemistry Department, Faculty of Chemistry and High Technologies, Kuban State University, Krasnodar 350040, Russia; abakumovleks@gmail.com (A.A.); khaliphyan@kubannet.ru (A.K.);; 2 Institute for Spectroscopy, Russian Academy of Sciences, Troitsk, Moscow 108840, Russia; mbolshov@mail.ru; 3 North Caucasian Federal Research Center of Horticulture, Viticulture, Wine–Making, Krasnodar 350072, Russia; 4 Institute for Geothermal Problems and Renewable Energy, Branch of the Joint Institute of High Temperatures of the Russian Academy of Sciences, Makhachkala 367030, Russia

**Keywords:** REE, wines, bentonites, elemental image, geographical origin and varietal image, discriminant analysis

## Abstract

The possibility of establishing the varietal and territorial affiliation of wines by the content of rare earth elements (REE) in them was studied. ICP–OES and ICP–MS with subsequent chemometric processing of the results were applied to determine the elemental image of soils containing negligible REE amounts, grapes grown on these soils, and wine materials of Cabernet Sauvignon, Merlot, and Moldova varieties produced from these grapes. To stabilize and clarify wine materials, the traditional processing of wine materials with various types of bentonite clays (BT) was used, which turned out to be a source of REE in the wine material. Discriminant analysis revealed that the processed wine materials were homogeneous within one denomination and that those of different denominations were heterogeneous with respect to the content of REE. It was found that REE in wine materials were transferred from BT during the processing, and thus they can poorly characterize the geographical origin and varietal affiliation of wines. Analysis of these wine materials according to the intrinsic concentrations of macro- and microelements showed that they formed clusters according to their varietal affiliation. In terms of their influence on the varietal image of wine materials, REE are significantly inferior to macro- and microelements, but they enhance their influence to a certain extent when used together.

## 1. Introduction

The confirmation of the quality and authenticity of dry wines and the control of the processes of their blending require the use of new analytical approaches allowing the consideration of wines as a whole based on the analysis of their “images”. In this case, the identification of the origin of wines is controlled by the characteristic components or elemental profiles (electrophoretic, chromatographic, and elemental) inherent to the studied sample of wine [1,2,3,4]. Due to the specificity of the obtained profiles, this approach makes it possible to determine the quality and affiliation of wines, to detect counterfeit products, and to identify wines by variety and origin [5,6,7].

In fact, the mineral composition is one of the main indicators of the classification of the varietal and geographical affiliation of wines due to the stability of the elemental composition of soils of grapes growing [8,9]. The relationship between the mineral composition in the soil–grape–wine chain makes it possible to establish the varietal and geographical origin of wine with high reliability due to the stability and specificity of the elemental composition of the grape variety cultivated in a particular area [8,9,10].

In many studies, rare earth elements (REE) have been considered as markers of the varietal and geographical origin of wines [11,12,13,14,15,16,17]. Many authors have used REE contents to distinguish wines from different regions of Italy [18,19], Spain [20], France [21], Germany [22], the USA [21,23], Australia [24], and South America [25].

The possibility of using REE as varietal markers based on the relationship between the contents of these elements in soil samples and various parts of grapes (berries, juice, leaves, seeds, and skins) was shown in [12]. Due to the different absorption of REE by the grape from volcanic soils characterized by a high content of these metals, each wine sample was found to belong to a particular grape variety. In Spain, 7 out of 12 studied regions of grape growth were identified by their REE concentrations [16]. The remaining regions, which were not properly differentiated, were additionally characterized taking into account the contents of Ba, Co, Cr, Mn, Ni, Pb, and V. Based on the contents of 31 elements, including REE, a model was obtained by chemometric methods, allowing the differentiation of samples of red wines of the Chianti region from other Italian regions [14].

Most of the above studies are aimed at studying the finished product, wine, which makes it difficult to establish its geographical origin [26,27,28]. Ideally, the elemental composition of wine should depend only on the mineral composition of the soil, grapes, and climatic conditions of its cultivation and not on the technology of production, transportation, or storage. However, the relationship in the soil–grape–wine chain is broken due to the fact that the elemental composition of wine is significantly affected by technological methods of their production, for example, their stabilization and clarification with auxiliary materials to remove an excess of components forming a haze of a various nature [10,26,27,28,29].

Magdas et al. [17] considered the possibilities and problems of using REE for the estimation of the quality of products, while they noted the low efficiency of REE applications in identifying wines. The limitation of using REE as a geographic marker is mainly due to the changes in the elemental composition during the production of wines. The authors of [13,22,30] noted that there was a significant increase in the concentrations of REE in wine materials at the stage of stabilization and clarification. It was noted [18,31] that very often, REE were absent in wine materials produced in different territories or their concentrations were negligible; therefore, the use of REE as markers of the geographical and varietal origin requires specific investigations for different regions of wine production. The only obvious fact is that the elemental image of wine, including the content of REE, can be significantly changed at various stages of wine production.

The purpose of this work is to assess the possibility of using REE as markers of the geographical origin, variety, formation of the cluster structure, and elemental image of wines. The paper presents the data of ICP–OES and ICP–MS studies of the elemental image of soils, grapes grown on soils, and wines of Cabernet Sauvignon, Merlot, and Moldova varieties produced from these grapes. All investigated wines were processed with bentonite clays (BT) at the stage of wine material stabilization and clarification. A total of 32 samples of BT of various compositions and from different manufacturers was used during the analysis of wine production.

## 2. Results and Discussion

A comparison of the elemental compositions of BT (Table 1) and tested wine materials (Table 2) proves that REE are introduced in the wine materials during the processing of rare materials by BT. The levels of almost all REE in the studied untreated wine materials were below the limits of quantification (LOQ), despite the high sensitivity of the spectrometer [32] (Table 2), although their contents in the final products were reliably recorded. It seemed important to determine the sources of REE entry into the analyzed wines and to establish a possible correlation of their contents with the quality of soils in the grape-growing region. The presence of Y, La, and Ce in untreated wine materials (Table 2) may be associated with anthropogenic impact, namely the introduction of soil improvers, fertilizers, pesticides, air pollution, or irrigation water [27,33]. 

**Figure 1:**
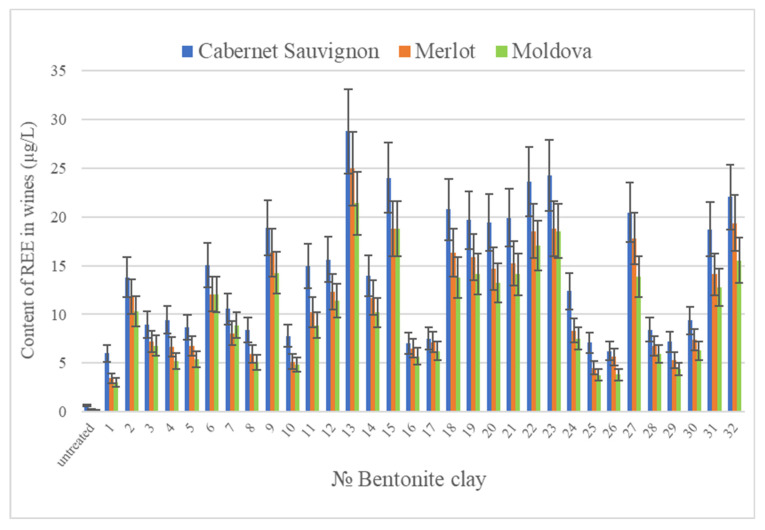
The total REE content in wine after BT treatment.

The total content of REE in the BT samples combined by groups differs (Table 1). The average minimum content of the total REE is observed in the BT samples of the fourth (7.93 μg/g), then the second (10.03 μg/g), the first (11.83 μg/g), and the third (12.32 μg/g) groups. BT significantly increases the content of REE in wine materials and there is a multiple increase in the concentrations of Ce (4–6), Y (2–19), and La (2–24) relative to their initial content in the wine materials, probably due to ion exchange (Table 2). The concentrations of Tm and Lu in the wine material of the Cabernet Sauvignon variety, Pr, Ho, Tm, and Lu in Merlot and Pr, Tb, Ho, and Tm in Moldova change the least. Notably, the greatest contribution of REE from BT to wine material is observed for the Cabernet Sauvignon variety, followed by Merlot and Moldova (Figure 1). The general nature of the increase in the content of REE in wine materials is quite similar—the greatest contribution to the increase in the content of REE in wine materials of all varieties is made by BT of the third (BT13, BT20, BT21, BT22, and BT27) and the first (BT19, BT23, and BT32) groups, as well as BT15 from the second group, the smallest—BT1 (Figure 1).

Table 3 shows the total contents of REE and trace elements (Ag, Al, As, Ba, Be, Bi, Cd, Co, Cs, Cu, Fe, Gd, Ge, Ho, Li, Mo, Mn, Nb, Ni, Pb, Ru, Sb, Sn, Sr, Ta, Th, Ti, Tl, U, V, W, Y, Zn, and Zr) after processing wine materials by BT of various groups. More detailed data on the effect of stabilization and clarification by BT on the elemental image of various varieties of wine materials are given in the Supplementary Material to the article.

There is a notable fact of an increase in the concentrations of REE and microelements in wine materials after treatment by BT of all groups. In addition, the introduction of BT of the same group during the clarification of different varieties of wine materials affects their elemental image in different ways.

Maximal concentrations of Pr and Eu were observed in Cabernet Sauvignon wine after clarification by BT of the first group; after clarification by BT of the second group—Y, Dy, Ho, Er, Tm, Yb, and Lu; and after BT of the third group—La, Ce, Nd, Sm, Gd, and Tb. Maximal concentrations of Eu were observed in Merlot wine material clarified by BT of the first group; by BT of the second group—Y, Tb, Ho, Er Tm, Yb, and Lu; and by BT of the third group—La, Ce, Pr, Nd, Sm, Gd, and Dy. In the wine material of Moldova, the maximum concentrations of La, Ce, Pr, Eu, and Ho were found after clarification by BT of the first group; Y, Er, and Lu after clarification by BT of the second group; and La, Nd, Sm, Gd, Tb, Dy, and Yb—after clarification by BT of the third group. The smallest change in the elemental composition of REE in all wine materials was caused by the BT of the fourth group.

Possible relationships between REE concentrations in bentonites and processed wine materials were studied by canonical correlation analysis (Table 4). For all three varieties of wine materials, the canonical correlation coefficients R were close to 1 (R = 0.99), indicating a high relationship between the REE content in bentonites and processed wine materials. The pair correlation coefficients r between the concentrations of REE in BT and wines shown in the table indicate strong relationships (r > 0.75), which are statistically significant (*p* < 0.05), and the content of REE in processed wine materials is directly proportional to their content in BT.

The possible varietal difference/similarity of samples of processed Cabernet Sauvignon, Merlot, and Moldova wine materials by the REE content in them was studied by discriminant analysis (Table 5). The predictors (independent variables) of the discrimination model were REE concentrations in BT-treated wine materials (32 samples each) and the grouping (dependent) variable was the wine material variety. The main criterion for evaluating the effectiveness of discrimination is the Wilks’ Lambda value, the total value of which for discriminant analysis, taking into account all the variables involved, is presented in the upper part of Table 5. Opposite each of the variables is the Wilks’ Lambda value for analysis in the case when this variable is not used. The Partial Lambda value characterizes the Wilks’ Lambda ratio after and before adding the corresponding variable. The Partial Lambda characterizes the single contribution of the corresponding variable to the separating force of the model.

The proximity of the Wilks’ Lambda (0.169) to zero in the upper box of Table 5 indicates successful discrimination, showing that the processed wine materials form clusters according to their varietal affiliation. Such discrimination confirms that wine materials within one denomination are homogeneous and have similarities in terms of the REE content. At the same time, wine materials of different denominations are heterogeneous with respect to the content of REE in them. Based on the Wilks’ Lambda value, which is the result of the exclusion of the corresponding metal from the discrimination model, the metal contribution to the separation procedure can be assessed: the larger its value, the higher the contribution, and hence the role of this metal in the model. Table 5 shows the sequence of REE in descending order in terms of their contribution to the discrimination model and the formation of a varietal cluster structure of wine materials. It can be seen that the largest contribution to the formation of the varietal cluster structure of wine materials based on the REE content is provided by Yb, then Lu, Sm, etc., and the smallest contribution is provided by Ho. At the same time, the contribution of sx elements (Yb, Lu, Sm, Gd, Er, and Dy) to discrimination is statistically significant—the significance level of the Fisher *p*-test is less than 0.05 (highlighted in bold).

**Figure 2:**
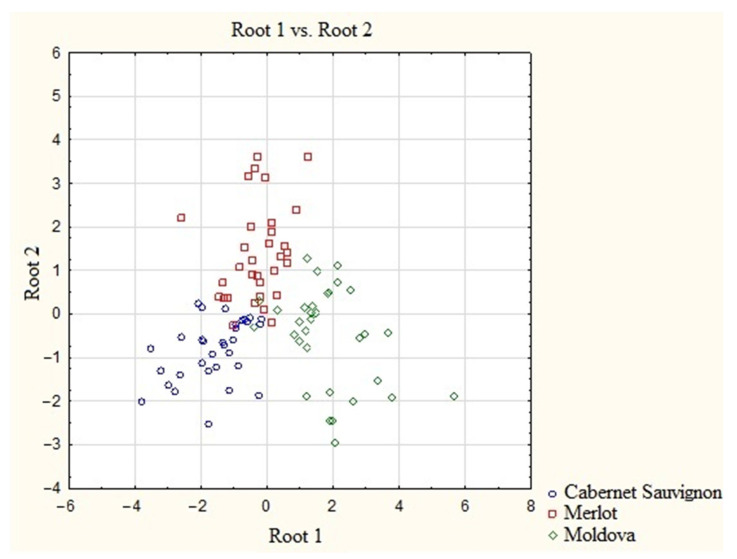
Scatter diagram of the canonical values of wine samples based on the concentrations of REE.

A graphical illustration of the presence of a varietal cluster structure is a scatterplot of canonical values (Figure 2) with the presentation of wine materials as points on a plane. The areas of grouping the canonical values of wine materials have different configurations and colors depending on the variety. This representation allows the visual assessment of the degree of similarity/difference between the varieties through distances according to the principle: the smaller the distance, the greater the similarity. The resulting graphic illustration shows that the distances between all varieties of wine materials are small despite the presence of a cluster structure, which means that inter-varietal differences are not sufficiently pronounced, especially between Cabernet Sauvignon and Merlot. Therefore, the use of REE as markers of the studied varieties of Kuban wine materials is problematic as it can lead to erroneous final results of their identification.

**Figure 3:**
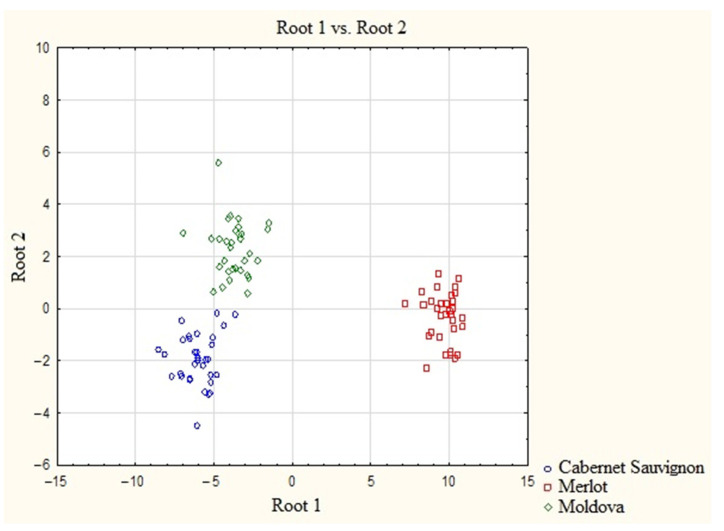
Scatter diagram of the canonical values of wine samples based on the concentrations of REE and marcoelements.

The cluster structure was significantly improved by adding macroelements, i.e., Ca, Mg, K, and Na, to the list of predictors in addition to REE as varietal markers of processed wine materials (Figure 3). In this case, the degree of homogeneity of the groups and the similarity between the cluster structures of wine materials improved. However, using the combination of REE and macroelements as varietal markers is insufficient for a correct analysis.

**Figure 4:**
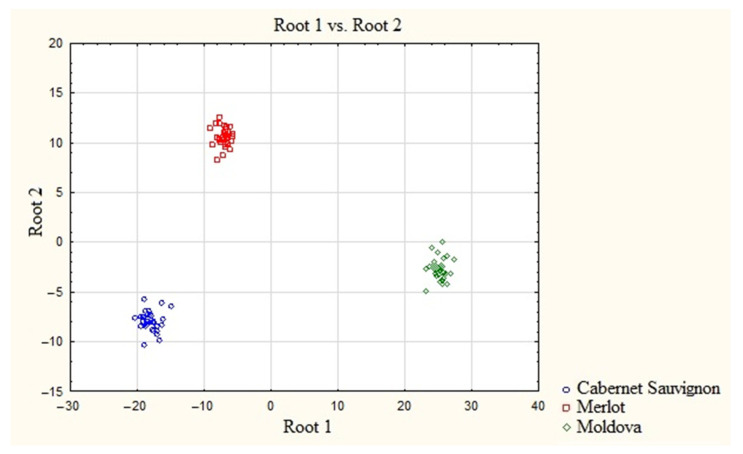
Scatter diagram of the canonical values of wine samples based on the concentrations of micro- and marcoelements [34].

In a previous work [34], we studied the role of macro elements, i.e., Ca, Mg, K, and Na, and microelements, i.e., Li, Co, Zr, Mo, Cd, Cu, Zn, Be, Ge, Nb, Rb, and Pb, in maintaining inter-varietal differences in wine materials. The combination of macro- and microelements led to a significant increase in intra-varietal similarity and inter-varietal differences in wine materials (Figure 4).

**Figure 5:**
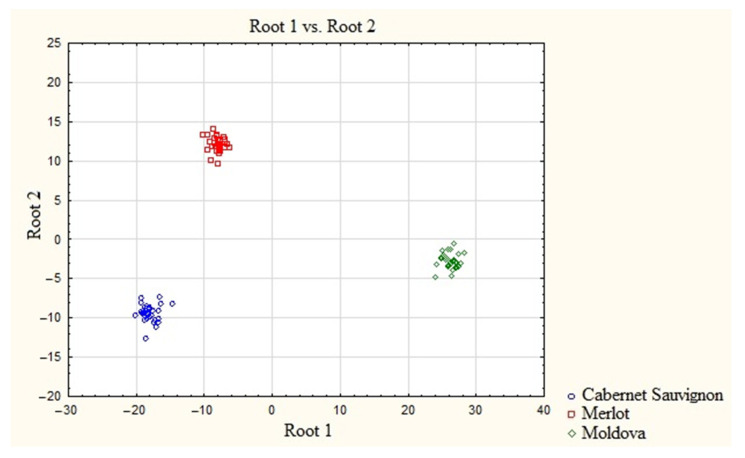
Scatter diagram of the canonical values of wine samples based on the concentrations of REE and micro- and marcoelements.

On the other hand, as a result of ion exchange, REE are included in the component composition of the wine material and, given the fact that their distribution is also different depending on the wine material variety, we considered their influence in maintaining inter-varietal differences in the composition with other elements. For this purpose, macro- and microelements were added to the REE predictors (Figure 5). Notably, the elemental images of wine materials after adding REE to the discrimination procedure retain the nature of the varietal difference in the elemental images of wine materials. The addition of REE had a slight effect on the elemental images of wine materials, increasing their intra-varietal similarity and inter-varietal differences. The weak influence of REE in the formation of the image of wine material is most likely due to the initial low content of REE in the soils of the grape-growing region.

## 3. Materials and Methods

### 3.1. Research Objects

The studies were carried out with wine materials produced from Cabernet Sauvignon, Merlot, and Moldova grape varieties. The grapes were harvested in September 2019 in the Temryuk region (Cabernet Sauvignon variety) and Krasnodar (Merlot and Moldova varieties) in the Krasnodar Territory, Russia. The processing of grapes and the production of wine materials were carried out in accordance with the general rules of the production of wines [35]. The procedure for processing grapes and obtaining wine materials is described in more detail in [29].

The stages of clarification and stabilization of wine materials by BT were studied using 32 samples of bentonite clays produced in different countries, with varying degrees of dispersion and trademarks (Table 6). All BT samples, except Khakass (BT13), Dagestan (BT15), and Crimean (BT27) origin are traditionally used in commercial wine-making technologies. Materials for claying wine materials (BT13, BT15, and BT27) were prepared from clays selected from deposits in the republics of Dagestan, Khakassia, and Crimea, considering the requirements for the production of wines [36].

In total, the studies were carried out with 99 samples from Cabernet Sauvignon, Merlot, and Moldova grape varieties, which included 3 initial and 96 samples of wine materials treated with bentonite clays (32 of each variety).

Inorganic Ventures IV-STOK-26 (USA) containing Ce, Dy, Er, Eu, Gd, Ho, La, Lu, Nd, Pr, Sm, Tb, Tm, Y, and Yb (10 mg/L of each element) were used to prepare calibration solutions. For the BT sample digestion, individual mineral acids were used: 15.4 mol/L HNO_3_, 25 mol/L HF, and 12 mol/L HCl (Sigma Aldrich, St. Louis, MO, USA). Solutions were prepared using deionized water (18.2 MΩ cm^−1^) obtained on a DuoPUR sub distillation unit (Milestone, Milan, Italy).

### 3.2. Procedures

#### 3.2.1. Wine Clarification and Stabilization Using BT

The clarification and stabilization of wine materials by BT was carried out according to the generally accepted technology in the scientific center “Winemaking” of the Federal Scientific Center for Horticulture, Viticulture, and Winemaking, Krasnodar, Russia [37]. A more detailed procedure for the clarification and stabilization of wine materials is described in [29].

#### 3.2.2. Elemental and X-ray Diffraction Analysis of BT

To study the possible effect of the procedure of clarification and stabilization by BT on the content of REE in wine materials, an X-ray phase analysis of the investigated fining agents was carried out on a Shimadzu XRD-7000 diffractometer (Shimadzu, Kyoto, Japan). According to the results of the X-ray phase analysis, the BT samples were divided into four groups, considering their qualitative and quantitative composition [29]. The first group included samples BT2, BT5, BT9, BT18, BT19, BT23, BT25, BT26, BT28, BT30, and BT32, which were based on sodium montmorillonite, the phase composition of which includes up to 3% of calcite (CaCO_3_). The second group included BT1, BT7, BT10, BT12, BT14, and BT15 with a base of sodium–calcium montmorillonite and up to 3% of quartz. The third group, i.e., BT3, BT4, BT6, BT8, BT13, BT16, BT17, BT20, BT21, BT22, BT24, BT27, and BT31, contained various forms of montmorillonite with quartz and calcite contents of more than 5%. The fourth group included BT11 and BT29. Sample BT11 was a mixture of sodium–calcium montmorillonite, 10% quartz, and BT29 in addition to sodium–calcium montmorillonite it contained approximately 4% of a non-clay mineral albite and silicon oxide in the form of cristobalite.

The elemental composition of BT was determined by inductively coupled plasma mass spectrometry (ICP–MS) on an iCAP RQ spectrometer (Thermo Scientific, Waltham, MA, USA). Sample introduction was performed using a borosilicate nebulizer MicroMist (Glass Expansion, Melbourne, Australia). The spray chamber was cooled to 2.8 °C by a Peltier element sample aerosol solution. The compromising balance between the sensitivity and minimization of the matrix effect was achieved by the construction of the spectrometer interface (Ni-sampler 1.1 mm diameter, Ni-scimmer 0.5 mm diameter, and 3.5 mm scimmer insert). Samples were prepared for analysis using a MARS 6 microwave system (CEM, Charlotte, NC, USA) considering the recommendations of the system manufacturer (digestion of clay) [38]. Then, 0.2 g of each BT sample was transferred to the vessel accompanying the microwave system employed and 5.0 mL of concentrated HF, 3.0 mL of concentrated HNO_3_, and 1.0 mL of concentrated HCl were added. The mixture in the flask was gradually heated up to 200 °C for 15 min, then kept at this temperature for 10 min. To eliminate the loss of volatile elements, the flasks were opened at a temperature below 40 °C, then the samples were transferred into 50 mL flasks and made up to the mark with deionized water.

#### 3.2.3. Determination of REE in Wines

The concentrations of REE in wine materials were determined by ICP–MS. A 10-fold dilution of wine samples with deionized water was chosen taking into account the literature data on their multi-element composition and the sensitivity of the analytical instruments used [39,40,41].

Considering the possible REE contents in wine materials, calibration curves were constructed using a set of standard solutions of Y, La, Ce, Pr, Nd, Sm, Eu, Gd, Tb, Dy, Ho, Er, Tm, Yb, and Lu with the analyte concentrations in a 0.001–20 µg/L range. The analysis conditions and operating parameters of the spectrometers are given in Table 7. The procedure for determining macro- and microelements in wine materials by the ICP–OES method is described in [29].

#### 3.2.4. Statistical Analysis

The relationship between the content of REE in BT and processed wine materials was established by correlation analysis. The degree of similarity/difference between samples of processed and unprocessed wine materials in terms of the REE content was assessed by discriminant analysis. The calculations were carried out using the STATISTICA program (v.13) [42].

## 4. Conclusions

The discriminant analysis of the elemental composition of wine materials obtained from grape varieties grown on soils practically free of REE has confirmed that they are introduced during the processing of wine materials and poorly characterize their varietal and regional affiliation. The resulting graphic illustration shows that, despite the presence of a cluster structure, the distances between all varieties of wine materials are small, which means that inter-varietal differences are not sufficiently pronounced, especially between Cabernet Sauvignon and Merlot. The processed wine materials within one denomination were homogeneous, and the wine materials of different denominations were heterogeneous in terms of their REE content. However, these differences are not sufficient for a reliable classification of wines based on the content of REE.

Therefore, the use of REE as markers of wine material varieties obtained from grape varieties grown on soils practically free of REE is problematic since it can lead to erroneous final results of their identification. In these cases, REE are significantly inferior to macro- and microelements in terms of their ability to represent the varietal image of wine materials.

It can be assumed that the use of ICP–OES and ICP–MS in combination with modern chemometric methods will reliably determine the quality and affiliation of wines by their elemental image and the relationship of components, detect counterfeits, and identify them by variety and origin.

## Figures and Tables

**Table 1:** REE content in BT samples, grouped based on their qualitative and quantitative analysis (N = 3, *p* = 0.95).

Element	Concentration, µg/g											
	Group 1			Group 2			Group 3			Group 4		
	Minimum	Average	Maximum	Minimum	Average	Maximum	Minimum	Average	Maximum	Minimum	Average	Maximum
Y	0.84	2.43	4.31	0.55	1.15	1.86	0.54	1.34	3.21	0.64	0.91	1.17
La	0.73	1.12	1.91	0.37	1.64	3.06	0.78	1.67	2.83	0.37	0.52	0.67
Ce	1.25	3.28	7.19	0.91	3.69	10.16	0.91	3.82	7.70	2.14	3.35	4.56
Pr	0.07	0.42	1.24	0.18	0.57	1.48	0.21	0.63	1.89	0.10	0.35	0.59
Nd	0.32	1.39	2.57	0.34	0.79	1.50	0.41	1.77	3.08	0.44	0.99	1.53
Sm	0.05	0.46	1.06	0.12	0.44	1.03	0.07	0.49	1.20	0.07	0.30	0.52
Eu	0.03	0.16	0.68	0.04	0.12	0.37	0.01	0.16	0.66	0.02	0.08	0.13
Gd	0.19	0.69	1.68	0.06	0.45	0.94	0.10	0.74	2.14	0.08	0.50	0.91
Tb	0.01	0.10	0.38	0.01	0.04	0.08	0.04	0.09	0.37	0.01	0.05	0.08
Dy	0.05	0.47	1.62	0.13	0.26	0.36	0.06	0.49	1.53	0.18	0.23	0.28
Ho	0.01	0.14	0.49	0.07	0.16	0.40	0.03	0.13	0.31	0.08	0.09	0.10
Er	0.10	0.33	0.85	0.10	0.26	0.37	0.04	0.24	0.58	0.14	0.14	0.14
Tm	0.01	0.09	0.19	0.01	0.03	0.06	0.01	0.06	0.17	0.01	0.01	0.01
Yb	0.21	0.61	1.13	0.24	0.37	0.62	0.08	0.57	1.29	0.30	0.35	0.40
Lu	0.02	0.14	0.31	0.01	0.06	0.15	0.01	0.12	0.35	0.01	0.06	0.11
Total	3.89	11.83	25.61	3.14	10.03	22.44	3.30	12.32	27.31	4.59	7.93	11.2

**Table 2:** REE concentration in wines before and after treatment with bentonite clays, µg/L (N = 3, *p* = 0.95).

REE	REE Content in Wine											
	Cabernet Sauvignon				Merlot				Moldova			
	Untreated	After BT Treatment			Untreated	After BT Treatment			Untreated	After BT Treatment		
		Minimum	Average	Maximum		Minimum	Average	Maximum		Minimum	Average	Maximum
Y	0.29 ± 0.04	0.64	2.16	5.54	0.11 ± 0.02	0.54	1.65	4.31	0.07 ± 0.01	0.47	1.97	4.97
La	0.18 ± 0.03	0.64	1.81	4.27	0.05 ± 0.01	0.37	1.40	3.06	0.05 ± 0.01	0.20	1.24	2.72
Ce	0.19 ± 0.03	1.55	4.51	12.36	0.08 ± 0.01	0.91	3.58	10.12	0.04 ± 0.01	0.75	2.99	9.50
Pr	<LOQ	0.12	0.66	2.55	<LOQ	<LOQ	0.53	1.89	<LOQ	<LOQ	0.37	1.25
Nd	<LOQ	0.34	1.75	3.84	<LOQ	0.32	1.41	3.08	<LOQ	0.22	0.99	2.33
Sm	<LOQ	0.09	0.51	1.56	<LOQ	<LOQ	0.46	1.20	<LOQ	0.07	0.41	1.23
Eu	<LOQ	0.07	0.17	0.52	<LOQ	0.01	0.15	0.68	<LOQ	<LOQ	0.12	0.36
Gd	<LOQ	0.20	0.95	2.41	<LOQ	0.06	0.65	2.14	<LOQ	0.05	0.51	1.28
Tb	<LOQ	0.04	0.14	0.42	<LOQ	<LOQ	0.08	0.38	<LOQ	<LOQ	0.04	0.16
Dy	<LOQ	0.09	0.56	1.99	<LOQ	0.05	0.42	1.62	<LOQ	0.03	0.31	0.93
Ho	<LOQ	0.05	0.19	0.67	<LOQ	<LOQ	0.11	0.49	<LOQ	<LOQ	0.14	0.63
Er	<LOQ	0.10	0.32	0.87	<LOQ	0.04	0.27	0.85	<LOQ	0.10	0.44	1.08
Tm	<LOQ	0.05	0.11	0.22	<LOQ	<LOQ	0.06	0.19	<LOQ	<LOQ	0.06	0.18
Yb	<LOQ	0.10	0.37	0.88	<LOQ	0.08	0.53	1.29	<LOQ	0.03	0.33	0.87
Lu	<LOQ	0.04	0.14	0.33	<LOQ	<LOQ	0.10	0.35	<LOQ	0.04	0.24	0.70

**Table 3:** Dynamics of changes in the total content of rare earth elements and microelements in various varieties of wines during treatment with BT.

Element	Concentration Range of Elements, mg/L				
	Untreated Wine	Group 1	Group 2	Group 3	Group 4
**Cabernet Sauvignon**					
REE, µg/L	0.66 ± 0.10	6.5–24.5	6.1–24.3	7.1–29.1	7.3–15.2
Microelements, mg/L	3.9 ± 0.6	4.1–6.5	4.2–6.0	4.2–6.1	4.1–4.6
**Merlot**					
REE, µg/L	0.24 ± 0.04	4.7–19.5	3.5–19.0	6.0–25.0	5.5–10.4
Microelements, mg/L	3.8 ± 0.6	4.2–6.6	4.0–5.8	4.1–6.1	4.1–4.7
**Moldova**					
REE, µg/L	0.16 ± 0.02	4.1–19.0	3.3–19.7	5.4–22.2	4.7–9.4
Microelements, mg/L	5.1 ± 0.8	5.5–8.0	5.1–7.4	5.3–7.8	5.4–6.0

**Table 4:** Correlation coefficients (R) between the concentrations of elements in bentonite clays and treated wines.

Element	Cabernet Sauvignon	Merlot	Moldova
Y	0.876	0.841	0.902
La	0.831	0.863	0.899
Ce	0.893	0.866	0.845
Pr	0.898	0.891	0.898
Nd	0.913	0.894	0.895
Sm	0.855	0.847	0.853
Eu	0.820	0.803	0.826
Gd	0.966	0.804	0.780
Tb	0.911	0.901	0.874
Dy	0.902	0.901	0.920
Ho	0.934	0.902	0.940
Er	0.904	0.870	0.907
Tm	0.896	0.920	0.845
Yb	0.952	0.902	0.919
Lu	0.869	0.902	0.924

**Table 5:** Results of the discriminant analysis of wine samples.

N = 96	Discriminant Function Analysis Summary No. of Vars in Model: 15; Grouping: Sort (3 grps) Wilks’ Lambda: 0.169; Approx. F (30.158) = 7.55, *p* < 0.05					
	Wilks’ Lambda	Partial Lambda	F-Remove (2.78)	*p*-Value	Tolerance	1-Tolerance (R-Sqr.)
**Yb**	**0.233**	**0.726**	**14.903**	**0.000**	**0.285**	**0.715**
**Lu**	**0.208**	**0.811**	**9.214**	**0.000**	**0.287**	**0.713**
**Sm**	**0.190**	**0.889**	**4.923**	**0.010**	**0.136**	**0.864**
**Gd**	**0.190**	**0.889**	**4.919**	**0.010**	**0.418**	**0.582**
**Er**	**0.189**	**0.895**	**4.651**	**0.012**	**0.247**	**0.753**
**Dy**	**0.186**	**0.906**	**4.098**	**0.020**	**0.238**	**0.762**
Tb	0.181	0.935	2.746	0.070	0.155	0.845
La	0.180	0.935	2.724	0.072	0.438	0.562
Ce	0.180	0.936	2.691	0.074	0.242	0.758
Tm	0.180	0.936	2.690	0.074	0.172	0.828
Y	0.179	0.941	2.462	0.092	0.314	0.686
Nd	0.171	0.985	0.616	0.543	0.334	0.666
Pr	0.171	0.985	0.602	0.550	0.155	0.845
Eu	0.171	0.986	0.545	0.582	0.263	0.737
Ho	0.170	0.991	0.377	0.687	0.318	0.682

**Table 6:** The list of studied and used BT.

No	BT Name	Country	Lightening Quality *	No	BT Name	Country	Lightening Quality
BT1	Electra	Italy	+++	BT17	BentoVinumGold (particle size 0.05 mm)	Kazakhstan	+++
BT2	Azerbaijan	Azerbaijan	++	BT18	Bentovin (particle size 0.07 mm)	Azerbaijan	+++
BT3	Claris P	Bosnia and Herzegovina	++	BT19	Bentovin (particle size 0.05 mm)	Azerbaijan	++
BT4	ClarisP70	Bosnia and Herzegovina	++	BT20	Vinobent field “10 Khutor” (particle size 0.07 mm)	Russia	++
BT5	GranuBent Pore-Tec	Germany	+++	BT21	Vinobent field “10 Khutor” (particle size 0.05 mm)	Russia	++
BT6	Aktivit	Germany	cloudy wine	BT22	Vinobent field “10 Khutor” production lot	Russia	++
BT7	Ca-Granulat	Germany	cloudy wine	BT23	Bentovin production lot	Azerbaijan	++
BT8	NaCalitPore-Tec	Germany	++	BT24	KaliNat Erbslöh	Germany	+
BT9	Gumbrin	Georgia	++	BT25	Aktivit Erbslöh	Germany	+++
BT10	Granula	France	+++	BT26	Extrabent	France	+++
BT11	Askangel	Georgia	+	BT27	Crimean bentonite	Russia	++
BT12	Ijevan bentonite	Armenia	+	BT28	Inobent	France	++
BT13	Khakass field	Russia	++	BT29	Seporit Pore-Tec	Germany	+++
BT14	Khakassia Sigma-Trade	Russia	++	BT30	Extrabent Super	France	+++
BT15	Dagestan field	Russia	++	BT31	ClarisP70 «Meridian»	Bosnia and Herzegovina	+
BT16	BentoVinumGold (particle size 0.07 mm)	Kazakhstan	+++	BT32	Kurtsevskoe field	Russia	+

* +++—crystal clear; ++—clear; +—light opalescence.

**Table 7:** Instrument operating parameters and REE limits of quantification.

ICP–MS (iCAP RQ)		
Plasma gas flowrate, L/min		15.0
Nebulizer gas flowrate, L/min		1.0
Auxiliary gas flowrate, L/min		0.8
Applied power, W		1400
Integration time, s		0.01
Nebulizer type, sample rate		MicroMist concentric nebulizer, 0.4 mL/min
Isotopes, (LOQ *, µg/L)		
^89^Y, (0.001); ^139^La, (0.001); ^140^Ce, (0.001); ^141^Pr, (0.011); ^146^Nd, (0.014); ^152^Sm, (0.006); ^151^Eu, (0.003); ^157^Gd, (0.002);	^159^Tb, (0.005); ^163^Dy, (0.003); ^165^Ho, (0.006); ^166^Er, (0.003); ^169^Tm, (0.002); ^174^Yb, (0.002); ^175^Lu, (0.002);	Interference correction equations Sm = I(^152^Sm) − 0.012780 × I(^157^Gd) Yb = I(^174^Yb) − 0.005934 × I(^178^Hf)

* LOQ = 10σ_0_, where σ_0_ is the standard deviation of blank results.

## Data Availability

Data included in the article/Supplementary Material are referenced in the article.

## Supplementary Materials

The following supporting information can be downloaded at: https://www.mdpi.com/article/10.3390/molecules28114319/s1, Table S1: Content of REE in untreated and treated with bentonite clays wines.

## Sample Availability

Not available.
