# Supplementary material for: Can Rare Earth Elements Be Considered as Markers of the Varietal and Geographical Origin of Wines?
Source: Molecules. 2023 May 24;28(11):4319. doi: 10.3390/molecules28114319 (PMC10254485; doi:10.3390/molecules28114319)
Supplement: Supplementary file 1 [file molecules-28-04319-s001.zip › molecules-2358117-supplementary.pdf]

Table S1. Content of REE in untreated and treated with bentonite clays wines.

№	Wine sample	Element, µg/L														
		Y	La	Ce	Pr	Nd	Sm	Eu	Gd	Tb	Dy	Ho	Er	Tm	Yb	Lu
1	Cabernet Sauvignon untreated	0.29	0.18	0.19	<LOQ	<LOQ	<LOQ	<LOQ	<LOQ	<LOQ	<LOQ	<LOQ	<LOQ	<LOQ	<LOQ	<LOQ
2	Cabernet Sauvignon treated with bentonite	1.38	0.90	1.55	0.26	0.51	0.13	0.14	0.20	0.08	0.16	0.08	0.18	0.08	0.25	0.09
3	Cabernet Sauvignon treated with bentonite	4.86	1.31	2.73	0.28	1.97	0.27	0.13	0.39	0.10	0.42	<LOQ	0.59	0.12	0.51	0.14
4	Cabernet Sauvignon treated with bentonite	1.53	0.90	1.75	0.55	2.51	0.13	0.18	0.59	0.07	0.17	0.08	0.17	0.07	0.18	0.08
5	Cabernet Sauvignon treated with bentonite	1.06	1.21	1.61	0.64	2.26	0.14	0.12	1.85	0.06	0.09	0.07	0.10	0.06	0.10	0.06
6	Cabernet Sauvignon treated with bentonite	1.80	0.70	2.12	0.22	0.34	0.10	0.11	2.41	0.06	0.15	0.08	0.14	0.08	0.26	0.09
7	Cabernet Sauvignon treated with bentonite	4.33	1.61	4.88	0.28	1.08	0.28	0.16	0.51	0.10	0.45	0.25	0.39	0.10	0.52	0.12
8	Cabernet Sauvignon treated with bentonite	1.29	2.03	2.82	0.37	1.42	0.37	0.13	0.46	0.10	0.47	0.33	0.44	0.09	0.17	0.09
9	Cabernet Sauvignon treated with bentonite	1.39	1.91	2.64	0.65	0.44	0.12	0.11	0.25	0.07	0.21	0.09	0.21	0.08	0.15	0.10
10	Cabernet Sauvignon treated with bentonite	1.85	2.51	5.31	1.05	3.89	0.44	0.21	0.57	0.42	0.57	0.67	0.44	0.22	0.57	0.15
11	Cabernet Sauvignon treated with bentonite	2.29	1.10	1.67	0.36	0.64	0.25	0.12	0.35	0.07	0.29	0.09	0.22	0.06	0.22	0.07
12	Cabernet Sauvignon treated with bentonite	1.06	0.64	6.42	1.14	2.29	0.75	0.07	1.14	0.15	0.43	0.14	0.24	0.09	0.27	0.13
13	Cabernet Sauvignon treated with bentonite	1.60	1.10	6.70	0.65	2.03	0.48	0.17	0.98	0.12	0.52	0.36	0.36	0.09	0.36	0.11
14	Cabernet Sauvignon treated with bentonite	2.66	3.15	8.93	2.16	4.24	1.56	0.45	2.10	0.41	1.13	0.34	0.46	0.21	0.74	0.24
15	Cabernet Sauvignon treated with bentonite	1.80	3.87	3.04	0.42	1.55	0.41	0.21	0.76	0.11	0.45	0.17	0.40	0.12	0.47	0.15
16	Cabernet Sauvignon treated with bentonite	0.85	2.31	12.36	2.55	0.68	1.25	0.52	1.91	0.15	0.29	0.16	0.38	0.15	0.28	0.17

№	Wine sample	Element, µg/L														
		Y	La	Ce	Pr	Nd	Sm	Eu	Gd	Tb	Dy	Ho	Er	Tm	Yb	Lu
17	Cabernet Sauvignon treated with bentonite	0.64	1.86	2.51	0.41	0.37	0.10	0.07	0.52	0.05	0.11	0.05	0.10	0.05	0.10	0.09
18	Cabernet Sauvignon treated with bentonite	0.93	1.62	2.67	0.36	0.43	0.15	0.09	0.54	0.08	0.15	0.08	0.15	0.08	0.13	0.05
19	Cabernet Sauvignon treated with bentonite	4.16	1.77	6.89	0.70	2.98	0.70	0.22	0.88	0.20	0.76	0.25	0.37	0.22	0.41	0.26
20	Cabernet Sauvignon treated with bentonite	4.25	1.84	6.83	0.70	1.85	0.70	0.15	0.90	0.15	0.75	0.23	0.37	0.22	0.47	0.28
21	Cabernet Sauvignon treated with bentonite	1.64	2.50	5.33	0.87	2.85	1.28	0.15	1.82	0.25	1.03	0.34	0.32	0.18	0.69	0.19
22	Cabernet Sauvignon treated with bentonite	1.84	2.52	5.33	0.82	2.96	1.35	0.19	1.89	0.25	1.05	0.30	0.39	0.16	0.72	0.16
23	Cabernet Sauvignon treated with bentonite	1.68	2.80	8.12	0.93	3.07	1.54	0.23	2.05	0.27	1.07	0.33	0.35	0.17	0.82	0.22
24	Cabernet Sauvignon treated with bentonite	4.95	2.05	8.52	0.85	3.23	0.73	0.18	1.09	0.17	0.83	0.28	0.38	0.22	0.49	0.32
25	Cabernet Sauvignon treated with bentonite	1.59	2.42	4.73	0.32	1.02	0.22	0.10	0.62	0.08	0.32	0.12	0.31	0.08	0.34	0.10
26	Cabernet Sauvignon treated with bentonite	1.28	1.72	2.21	0.12	0.38	0.09	0.09	0.43	0.05	0.12	0.07	0.16	0.06	0.23	0.07
27	Cabernet Sauvignon treated with bentonite	1.18	0.72	2.19	0.12	0.57	0.11	0.07	0.47	<LOQ	0.33	0.06	0.12	0.05	0.18	0.07
28	Cabernet Sauvignon treated with bentonite	1.79	2.92	5.40	0.85	3.72	0.83	0.25	1.25	0.17	1.85	0.23	0.63	0.14	0.29	0.15
29	Cabernet Sauvignon treated with bentonite	1.27	1.30	2.54	0.23	0.76	0.17	0.11	0.54	0.06	0.65	0.09	0.24	0.07	0.32	0.09
30	Cabernet Sauvignon treated with bentonite	1.46	0.97	2.75	0.18	0.51	0.14	0.09	0.20	0.05	0.27	0.07	0.11	0.06	0.19	0.13
31	Cabernet Sauvignon treated with bentonite	1.79	1.12	2.70	0.55	1.45	0.20	0.14	0.45	0.10	0.33	0.08	0.13	0.09	0.23	0.02
32	Cabernet Sauvignon treated with bentonite	3.35	2.01	7.86	0.48	1.87	0.65	0.18	0.58	0.11	0.47	0.10	0.66	0.06	0.25	0.11
33	Cabernet Sauvignon treated with bentonite	5.54	2.50	3.27	0.89	2.12	0.78	0.29	1.74	0.25	1.99	0.48	0.87	0.12	0.88	

№	Wine sample	Element, µg/L														
		Y	La	Ce	Pr	Nd	Sm	Eu	Gd	Tb	Dy	Ho	Er	Tm	Yb	Lu
																0.33
34	Merlot untreated	0.11	0.05	0.08	<LOQ	<LOQ	<LOQ	<LOQ	<LOQ	<LOQ	<LOQ	<LOQ	<LOQ	<LOQ	<LOQ	<LOQ
35	Merlot treated with bentonite	0.79	0.37	1.04	0.18	0.35	0.12	0.06	0.06	<LOQ	0.13	<LOQ	0.10	<LOQ	0.24	<LOQ
36	Merlot treated with bentonite	3.25	1.45	2.60	0.23	1.44	0.34	0.14	0.25	0.05	0.30	0.09	0.53	0.02	0.97	0.13
37	Merlot treated with bentonite	0.79	1.05	1.28	0.42	2.46	0.07	0.15	0.47	<LOQ	0.14	<LOQ	0.07	<LOQ	0.31	<LOQ
38	Merlot treated with bentonite	0.54	1.50	0.91	0.46	2.12	0.20	0.14	0.66	<LOQ	0.06	<LOQ	0.04	<LOQ	0.08	<LOQ
39	Merlot treated with bentonite	1.52	0.73	1.88	<LOQ	0.32	<LOQ	0.03	1.68	<LOQ	0.12	<LOQ	0.10	<LOQ	0.40	<LOQ
40	Merlot treated with bentonite	3.21	0.78	3.98	0.22	0.92	0.18	0.06	0.83	<LOQ	0.37	0.24	0.26	0.03	0.87	0.14
41	Merlot treated with bentonite	1.03	1.71	1.97	0.52	0.93	0.24	0.04	0.51	<LOQ	0.33	0.20	0.32	<LOQ	0.26	<LOQ
42	Merlot treated with bentonite	1.09	1.45	1.32	0.59	0.51	0.08	0.04	0.10	<LOQ	0.17	<LOQ	0.12	<LOQ	0.26	0.20
43	Merlot treated with bentonite	1.32	1.91	4.03	1.09	2.28	0.57	0.68	1.19	0.38	0.46	0.49	0.39	0.18	1.13	0.21
44	Merlot treated with bentonite	1.86	0.68	0.91	0.25	0.34	0.24	0.04	0.22	<LOQ	0.14	<LOQ	0.18	<LOQ	0.27	<LOQ
45	Merlot treated with bentonite	0.64	0.37	4.56	0.59	1.53	0.52	<LOQ	0.91	0.08	0.28	0.10	0.14	<LOQ	0.40	0.11
46	Merlot treated with bentonite	1.24	1.55	5.77	0.39	0.91	0.48	0.05	0.43	<LOQ	0.36	0.40	0.28	0.04	0.38	0.04
47	Merlot treated with bentonite	2.30	2.83	7.70	1.89	2.67	0.95	0.66	2.14	0.37	0.93	0.31	0.39	0.17	1.29	0.35
48	Merlot treated with bentonite	1.40	3.06	2.26	0.61	1.50	0.54	0.15	0.56	0.08	0.36	0.10	0.37	0.06	0.62	0.06
49	Merlot treated with bentonite	0.55	2.48	10.16	1.48	0.68	1.03	0.37	0.94	<LOQ	0.21	<LOQ	0.31	<LOQ	0.46	0.15
50	Merlot treated with bentonite	0.56	1.58	2.70	0.35	0.41	0.07	<LOQ	0.53	<LOQ	0.10	<LOQ	0.06	<LOQ	0.15	<LOQ

№	Wine sample	Element, µg/L														
		**Y**	**La**	**Ce**	**Pr**	**Nd**	**Sm**	**Eu**	**Gd**	**Tb**	**Dy**	**Ho**	**Er**	**Tm**	**Yb**	**Lu**
51	Merlot treated with bentonite	0.66	1.36	3.07	0.32	0.52	0.13	0.04	0.52	0.05	0.11	<LOQ	0.07	0.03	0.23	0.04
52	Merlot treated with bentonite	3.65	0.81	5.58	0.40	1.95	1.06	0.11	0.62	0.13	0.58	0.14	0.35	0.17	0.54	0.22
53	Merlot treated with bentonite	3.80	0.88	5.66	0.36	1.32	0.91	0.07	0.64	0.10	0.61	0.13	0.37	0.16	0.60	0.25
54	Merlot treated with bentonite	0.97	1.70	4.44	0.56	2.70	0.83	0.14	0.85	0.11	0.75	0.15	0.27	0.11	0.96	0.13
55	Merlot treated with bentonite	1.17	1.73	4.37	0.64	3.01	0.80	0.17	0.87	0.13	0.81	0.16	0.25	0.09	0.94	0.13
56	Merlot treated with bentonite	1.37	1.95	6.19	0.84	3.08	1.19	0.19	0.90	0.16	0.85	0.21	0.27	0.13	1.07	0.15
57	Merlot treated with bentonite	4.31	0.93	7.19	0.49	2.12	0.53	0.06	0.76	0.07	0.63	0.15	0.37	0.19	0.70	0.31
58	Merlot treated with bentonite	0.96	1.41	3.69	0.27	0.61	0.21	<LOQ	0.41	<LOQ	0.14	<LOQ	0.20	<LOQ	0.45	<LOQ
59	Merlot treated with bentonite	1.09	0.85	1.25	<LOQ	0.45	0.08	0.06	0.26	<LOQ	0.05	<LOQ	0.12	<LOQ	0.27	0.02
60	Merlot treated with bentonite	1.03	0.79	1.87	0.16	0.67	0.09	0.06	0.33	<LOQ	0.24	<LOQ	0.17	<LOQ	0.21	0.02
61	Merlot treated with bentonite	0.88	2.64	4.10	1.44	2.81	1.20	0.37	1.03	0.23	1.53	0.28	0.52	0.14	0.46	0.15
62	Merlot treated with bentonite	0.84	0.82	2.07	0.21	1.05	0.21	0.07	0.27	<LOQ	0.40	<LOQ	0.23	0.04	0.49	0.05
63	Merlot treated with bentonite	1.17	0.67	2.14	<LOQ	0.44	0.07	0.13	0.08	<LOQ	0.18	<LOQ	0.14	<LOQ	0.30	<LOQ
64	Merlot treated with bentonite	1.65	1.32	1.93	0.26	1.09	0.14	0.06	0.19	<LOQ	0.21	<LOQ	0.15	0.02	0.34	0.02
65	Merlot treated with bentonite	2.89	1.70	5.93	0.21	1.22	0.46	0.12	0.29	<LOQ	0.35	<LOQ	0.58	0.02	0.31	0.02
66	Merlot treated with bentonite	4.28	1.81	2.04	1.24	2.57	1.05	0.44	1.44	0.24	1.62	0.33	0.85	0.19	1.05	0.23
67	Moldona untreated	0.07	0.05	0.04	<LOQ	<LOQ	<LOQ	<LOQ	<LOQ	<LOQ	<LOQ	<LOQ	<LOQ	<LOQ	<LOQ	<LOQ

№	Wine sample	Element, µg/L														
		Y	La	Ce	Pr	Nd	Sm	Eu	Gd	Tb	Dy	Ho	Er	Tm	Yb	Lu
68	Moldova treated with bentonite	0.88	0.20	0.75	<LOQ	0.33	0.10	0.11	0.05	<LOQ	0.13	0.06	0.20	0.05	0.13	0.04
69	Moldova treated with bentonite	4.31	1.15	1.57	0.15	0.56	0.23	0.11	0.08	<LOQ	0.26	<LOQ	0.78	0.06	0.58	0.45
70	Moldova treated with bentonite	1.61	0.81	1.08	0.30	1.59	0.16	0.14	0.34	<LOQ	0.16	<LOQ	0.29	0.02	0.17	0.11
71	Moldova treated with bentonite	0.52	1.26	0.81	0.30	1.40	0.10	0.13	0.45	<LOQ	0.03	<LOQ	0.10	0.02	0.03	0.08
72	Moldova treated with bentonite	1.86	0.69	1.06	<LOQ	0.42	0.10	0.04	0.61	<LOQ	0.08	<LOQ	0.20	0.02	0.26	0.08
73	Moldova treated with bentonite	4.49	0.72	2.98	0.17	0.91	0.33	0.12	0.37	0.07	0.29	0.21	0.49	0.02	0.59	0.27
74	Moldova treated with bentonite	1.73	1.96	1.71	0.33	0.94	0.40	0.10	0.25	<LOQ	0.18	0.32	0.69	0.03	0.13	0.11
75	Moldova treated with bentonite	1.06	1.08	1.09	0.49	0.22	0.07	0.10	0.05	<LOQ	0.08	<LOQ	0.33	0.02	0.16	0.34
76	Moldova treated with bentonite	1.67	1.84	3.62	0.73	2.08	0.53	0.24	0.52	0.16	0.41	0.63	0.79	0.10	0.53	0.42
77	Moldova treated with bentonite	1.95	0.59	0.79	0.14	0.23	0.17	0.03	0.18	<LOQ	0.11	0.06	0.30	<LOQ	0.19	0.08
78	Moldova treated with bentonite	0.91	0.33	3.69	0.47	1.14	0.33	<LOQ	0.82	<LOQ	0.20	0.06	0.41	<LOQ	0.23	0.30
79	Moldova treated with bentonite	1.72	0.90	5.04	0.30	1.03	0.43	0.08	0.50	<LOQ	0.26	0.31	0.39	0.05	0.29	0.08
80	Moldova treated with bentonite	3.73	2.68	6.29	1.03	2.33	1.10	0.34	0.82	0.11	0.73	0.31	0.65	0.13	0.86	0.29
81	Moldova treated with bentonite	1.23	2.72	2.10	0.53	1.36	0.23	0.16	0.47	<LOQ	0.21	0.18	0.53	0.04	0.33	0.11
82	Moldova treated with bentonite	0.92	2.29	9.50	1.25	0.51	1.23	0.36	1.21	0.05	0.16	0.15	0.48	0.05	0.23	0.38
83	Moldova treated with bentonite	0.47	1.41	2.37	0.33	0.33	0.07	<LOQ	0.37	<LOQ	0.06	<LOQ	0.10	0.03	0.08	0.08
84	Moldova treated with bentonite	0.65	1.34	2.53	0.34	0.48	0.19	0.04	0.35	<LOQ	0.05	<LOQ	0.10	<LOQ	0.13	0.04

№	Wine sample	Element, µg/L														
		Y	La	Ce	Pr	Nd	Sm	Eu	Gd	Tb	Dy	Ho	Er	Tm	Yb	Lu
85	Moldova treated with bentonite	3.69	0.97	4.32	0.33	1.04	0.53	0.10	0.66	0.05	0.47	0.24	0.46	0.14	0.29	0.49
86	Moldova treated with bentonite	3.95	1.01	4.62	0.32	0.89	0.51	0.09	0.61	<LOQ	0.47	0.20	0.47	0.18	0.28	0.56
87	Moldova treated with bentonite	1.20	1.64	3.89	0.49	1.53	0.66	0.08	1.18	<LOQ	0.79	0.18	0.49	0.07	0.69	0.33
88	Moldova treated with bentonite	1.39	1.54	4.03	0.52	1.60	1.12	0.06	1.15	0.06	0.89	0.21	0.44	0.08	0.68	0.33
89	Moldova treated with bentonite	1.67	1.78	5.64	0.61	1.71	1.18	0.15	1.28	0.10	0.93	0.24	0.46	0.13	0.82	0.37
90	Moldova treated with bentonite	4.97	1.21	6.18	0.45	1.82	0.56	0.16	0.74	0.06	0.46	0.24	0.52	0.17	0.38	0.61
91	Moldova treated with bentonite	1.22	1.32	2.66	0.26	0.41	0.26	0.04	0.38	<LOQ	0.08	0.07	0.39	0.04	0.30	0.08
92	Moldova treated with bentonite	1.22	0.33	1.01	<LOQ	0.34	0.07	0.10	0.21	<LOQ	0.05	<LOQ	0.29	<LOQ	0.13	0.04
93	Moldova treated with bentonite	0.70	0.40	1.25	0.14	0.41	0.07	0.04	0.28	<LOQ	0.08	<LOQ	0.28	0.03	0.06	0.07
94	Moldova treated with bentonite	1.44	2.07	3.58	0.64	1.52	0.66	0.17	0.95	0.12	0.93	0.16	0.82	0.07	0.43	0.33
95	Moldova treated with bentonite	1.09	0.63	1.42	0.33	0.58	0.33	0.09	0.35	<LOQ	0.25	<LOQ	0.35	0.02	0.40	0.12
96	Moldova treated with bentonite	1.26	0.49	1.19	<LOQ	0.48	0.20	0.20	0.02	<LOQ	0.07	<LOQ	0.19	0.02	0.20	0.08
97	Moldova treated with bentonite	1.63	0.79	1.66	0.28	0.85	0.31	0.09	0.15	<LOQ	0.08	<LOQ	0.20	0.07	0.06	0.08
98	Moldova treated with bentonite	2.50	1.67	5.85	0.15	0.76	0.33	0.16	0.21	<LOQ	0.16	<LOQ	0.79	0.03	0.10	0.08
99	Moldova treated with bentonite	4.30	1.88	1.53	0.32	1.73	0.63	0.28	0.73	0.08	0.91	0.37	1.08	0.14	0.85	0.70
